# Accuracy of site versus core laboratory interpretations of right heart catheterization hemodynamics

**DOI:** 10.1016/j.ahj.2026.107351

**Published:** 2026-01-12

**Authors:** Omar Cantu-Martinez, Marat Fudim, David F. Kong, Ryan J. Tedford, Philip G. Jones, Andrew J. Sauer, Timothy J. Fendler, John A. Spertus

**Affiliations:** a Kansas City’s Healthcare Institute for Innovations in Quality, University of Missouri, Kansas City, MO; b Saint Luke’s Mid America Heart Institute, Kansas City, MO; c Duke Clinical Research Institute, Durham, NC; d Medical University of South Carolina, Charleston, SC

## Abstract

An accurate interpretation of hemodynamics measured by right heart catheterization (RHC) is crucial for guiding the management of heart failure and pulmonary hypertension. We evaluated the concordance and variability between site-reported and core laboratory-adjudicated interpretations of invasive hemodynamic measurements.

## Background

Right heart catheterization (RHC) is crucial for informing diagnosis and treatment decisions in heart failure (HF) and pulmonary hypertension (PH).^1 ,2^ Current guidelines encourage research into noninvasive remote hemodynamic monitoring, while recognizing invasive RHC as the gold standard.^1^ Because the accuracy of RHC interpretations has not been rigorously assessed, we evaluated the concordance and variability of invasive hemodynamic data interpretation between clinical sites and a core laboratory (core lab).

## Methods

A descriptive analysis was performed using deidentified data (August 2023-October 2024) from *CAPTURE-HF* (Machine Learning Model’s Performance for Noninvasive Intracardiac Pressure Monitoring in HF) a prospective, multicenter validation study of a noninvasive pressure sensor in all-comer adults undergoing RHC, where sites performed initial interpretations that were subsequently adjudicated by the core lab. Noninvasive sensor signals collected for the parent study were not included in these analyses. The dataset provided by the sponsor (Acorai, Helsingborg, Sweden) included de-identified demographics, site-, and core lab-interpreted invasive RHC measurements of right atrial pressure, pulmonary capillary wedge pressure (PCWP), and pulmonary artery systolic, diastolic, and mean pressures (mPAP; calculated as 2/3 diastolic + 1/3 systolic).

Standard procedural recommendations provided by the sponsor included internal jugular or brachial venous access with transducers being leveled and zeroed at the mid-chest line with the catheter tip at the same height. Measurements were to be obtained during free breathing, as recommended by ISHLT guidelines and sites were advised to minimize artifacts, such as catheter ringing and dampened signals. PCWP was measured as the end-expiratory mean crossing the A-wave over 3 respiratory cycles without ectopy. For atrial fibrillation, averaging across respiratory cycles reduces variability from irregular R-R intervals.

Digitized hemodynamic waveform images from 20 sites were independently reviewed by 2 blinded core lab experts at the Duke Clinical Research Institute using standardized protocols and digital calipers (Iconico, Inc. Philadelphia, PA). While *CAPTURE-HF* obtained patient consent, the Saint Luke’s Institutional Review Board waived additional consent for this secondary analysis.

Analyses focused on PCWP and mPAP, given their clinical importance in assessing pulmonary congestion and PH. RHC measurements were summarized by median and interquartile range (IQR). Site and core lab interpretations were compared using Pearson correlation coefficients, Bland-Altman analyses, and empirical cumulative distribution function (ECDF) curves of site minus core lab differences. Due to non-normal distributions, median differences and nonparametric limits of agreement (LOA; 2.5th and 97.5th percentiles) were reported. Median regression lines were overlaid on Bland-Altman plots to assess systematic bias.^3^ Misclassification rates between sites and the core lab were assessed using clinically meaningful thresholds: PCWP ≥18 mmHg (pulmonary congestion), mPAP >20 mmHg (diagnosing PH), and PCWP >15 mmHg (postcapillary PH),^2^ for which under- and over-estimation would be clinically significant. Hierarchical linear models were fit to estimate the proportion of variability (R^2^) of site and core lab differences attributable to sites, operators, and selected patient characteristics (age, sex, body mass index, and RHC value).^4^ Exploratory analyses evaluated the association between body mass index and site versus core lab pressure differences.

## Results

Among 1560 patients (mean age 63.2 ± 14.3 years; body mass index 28.7 ± 6.7; 37% women), site-reported values had a median (IQR) of 13.0 (8.0-20.0) mmHg for PCWP and 24.3 (17.3-34.3) mmHg for mPAP. Core lab-adjudicated values were slightly higher at 14.0 (9.0-21.0) mmHg for PCWP and 27.0 (19.0-37.0) mmHg for mPAP.

**Figure 1.**
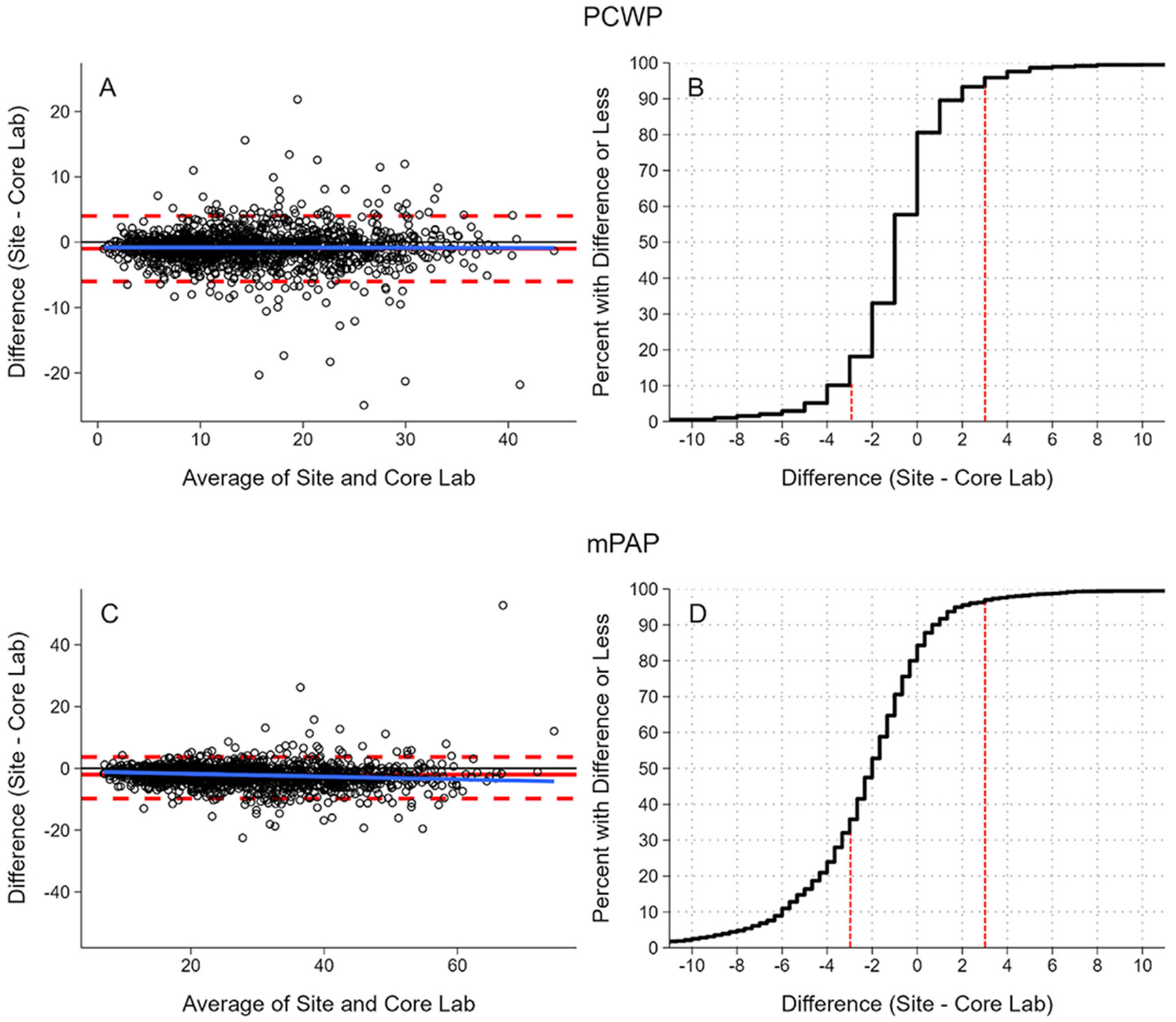
Bland-Altman and ECDF of site vs core lab hemodynamic reads of PCWP and mPAP. Abbreviations: ECDF, empirical cumulative distribution function; PCWP, pulmonary capillary wedge pressure; mPAP, mean pulmonary artery pressure. Panels A and C show Bland-Altman plots comparing site-reported and core lab-adjudicated measurements for PCWP and mPAP, respectively. The x-axis represents the average of site and core lab values, and the y-axis represents the difference (site minus core lab). The solid blue line indicates the median regression; horizontal red dashed lines mark the 2.5th and 97.5th percentiles. Panels B and D show ECDF curves for PCWP and mPAP, respectively. The x-axis represents the difference between site and core lab measurements (mmHg), where negative values indicate lower site measurements (underestimation) and positive values indicate higher site measurements (overestimation). The y-axis represents the cumulative proportion of patients with a difference of that magnitude or less. Vertical red dashed lines highlight example thresholds discussed in the text.

Correlations between site and core lab measurements were strong (*r* = 0.94 for PCWP; *r* = 0.96 for mPAP). Bland-Altman analysis showed a median difference of −1.0 mmHg for PCWP with 95% LOA −6.0 to 4.0 mmHg (Figure 1A), and −2.0 mmHg for mPAP with 95% LOA −9.8 to 3.7 mmHg (Figure 1C), indicating modest variability.

ECDF curves of site minus core lab differences for PCWP were small, tightly distributed, and centered near zero, with fewer than 10% of site-reported values ≥3 mmHg lower and fewer than 5% ≥3 mmHg higher than core lab-adjudicated values (Figure 1B). In contrast, differences in mPAP were more variable, with approximately 30% of site-reported values being ≥3 mmHg lower, while fewer than 5% were ≥3mmHg higher than core lab values (Figure 1D).

Table 1 summarizes misclassification rates at clinically relevant thresholds for diagnosing pulmonary congestion and PH. Underestimation occurred in 12.9% of cases for PCWP ≥18 mmHg, 9.6% for mPAP >20 mmHg, and 13.7% for PCWP >15 mmHg. Overestimation was less frequent, occurring in 3.2% of cases for PCWP ≥18 mmHg, 4.7% for mPAP >20 mmHg, and 4.5% for PCWP >15 mmHg.

Hierarchical linear models showed that variability in PCWP differences was minimally attributable to sites (R^2^ = 1.5%), operators (2.5%), or measured patient characteristics (3.8%), with most variation unexplained (92.2%). For mPAP, 9.8% of the variability in differences was explained by sites, 5.5% by operators, and 7.7% by patient characteristics, leaving 77.0% unexplained.

BMI was associated with slightly lower site- versus core lab-estimates for both PCWP (−0.29 mmHg per +5 kg/m^2^; 95% CI −0.40 to −0.18; *P <* .001) and mPAP (−0.37 mmHg per +5 kg/m^2^; 95% CI −0.51 to −0.24; *P <* .001). These effect sizes were small and consistent with the minimal variability in PCWP (3.8%) and mPAP (7.7%) differences explained by patient characteristics.

## Discussion

Understanding the accuracy of diagnostic procedures is critical for supporting high-quality clinical care, but is rarely assessed. In this study, correlation coefficients and median differences indicated strong overall agreement between site and core lab interpretations of RHC measurements. Nonetheless, notable patterns were observed. Site agreement with core lab interpretations was higher for PCWP than for mPAP, with a tighter distribution of PCWP differences that rarely exceeded ±3 mmHg and low rates of misclassification at clinically important PCWP thresholds (<13% underestimation, <5% overestimation). In contrast, site-reported mPAP was more variable, with approximately one-third of sites underestimating the core lab values by ≥3 mmHg, although misclassification rates around the 20 mmHg mPAP threshold were similar to those observed for PCWP. Despite observing only modest underestimation (~10%-13%), about a quarter of site interpretations could still have led to inappropriate clinical decisions, given that even small errors can affect recognition of congestion and PH classification. Overestimation was less frequent (<5% across thresholds) but could potentially lead to unnecessary diuresis or PH misclassification.

Similarly, while the Bland-Altman analysis showed a wide LOA, their clinical relevance must be weighed against the low misclassification rates. As a descriptive example, a core lab PCWP of 18 mmHg could correspond to site estimates ranging from 12 to 22 mmHg, and a core lab mPAP of 21 mmHg could correspond to site estimates between 11.2 and 24.7 mmHg. Despite high overall agreement between site and core lab hemodynamic measurements, such variability could also affect patient-level decisions and underscores the importance of accurate hemodynamic measurement and interpretation. Since this study focused upon measurements alone, and not the clinical actions performed, the impact of misclassification is not known and may be mitigated in real-world practice, where clinicians integrate hemodynamic data within a broader clinical context before altering treatments. Nevertheless, these findings highlight an important opportunity for sites to audit and improve the precision of RHC performance, when needed.

Although direct comparisons between routine site and core lab RHC interpretations are lacking, prior studies have documented variability in hemodynamic assessment across HF specialists, interventional cardiologists, and varying operator experience.^5^ Although literature suggests broad operator variability, we found only a small proportion of variance attributable to site or operator, with most being random. While reassuring that no systematic bias exists across sites were identified, reducing random variation remains an important tenet of quality improvement.

One potential strategy is to focus on technical factors inherent in invasive hemodynamic measurement. Accurate measurements require proper transducer leveling and zeroing at the mid-chest, high-fidelity waveforms with normal amplitude, a right ventricular end-diastolic inflection, a pulmonary artery dicrotic notch, and minimal artifacts. Overdamping, underdamping, or excessive catheter movement can alter estimates but are often corrected with flushing, repositioning, or balloon deflation.^6^ However, even when these factors are optimized, the real-world quality of waveform interpretation can vary and influence clinical decisions. Routine hemodynamic waveform quality and interpretation review in clinical practice may help reduce variability and facilitate a more consistent, holistic approach to decision-making.

### Limitations

First, invasive hemodynamic data were obtained from selected specialized sites participating in a device validation study, where core lab-imparted training and more attention to accuracy may have introduced a Hawthorne effect that reduced observed variability. Replicating these analyses in routine clinical settings are needed. Second, site-level procedural details were unavailable, but seem unlikely to explain the modest variability observed. Third, we could not assess treatment decisions or outcomes based on measurement discrepancies, undermining the ability to assess the clinical impact of inaccurate measurements. Fourth, cardiac output was not collected, preventing calculation of pulmonary vascular resistance that distinguishes pre from postcapillary PH. Finally, digitized waveforms may have impacted core lab fidelity, although centralized adjudication ensures consistency across sites, thereby increasing the rigor of this analysis.

## Conclusion

Although site- and core lab RHC assessments were highly concordant on average, approximately 1 in 4 patients could have been misclassified at clinically relevant thresholds. Given the diagnostic and therapeutic implications, sites should consider auditing their hemodynamic measurements for accuracy.

## Figures and Tables

**Table 1.** Misclassification of site vs core lab hemodynamic interpretations at clinically important thresholds.

Threshold	Underestimation	Overestimation	Total reads
PCWP ≥ 18 mmHg	69 / 535 (12.9%)	32 / 994 (3.2%)	1,529
mPAP > 20 mmHg	103 / 1,078 (9.6%)	22 / 469 (4.7%)	1,547
PCWP > 15 mmHg	88 / 643 (13.7%)	40 / 886 (4.5%)	1,529

Abbreviations: PCWP, pulmonary capillary wedge pressure; mPAP, mean pulmonary artery pressure.

## Abbreviations:

core lab: core laboratory
ECDF: empirical cumulative distribution function
HF: heart failure
IQR: interquartile range
LOA: limits of agreement
mPAP: mean pulmonary artery pressure
PCWP: pulmonary capillary wedge pressure
PH: pulmonary hypertension
RHC: right heart catheterization
